# Clinical management of respiratory syndrome in patients hospitalized for suspected Middle East respiratory syndrome coronavirus infection in the Paris area from 2013 to 2016

**DOI:** 10.1186/s12879-018-3223-5

**Published:** 2018-07-16

**Authors:** A. Bleibtreu, S. Jaureguiberry, N. Houhou, D. Boutolleau, H. Guillot, D. Vallois, J. C. Lucet, J. Robert, B. Mourvillier, J. Delemazure, M. Jaspard, F. X. Lescure, C. Rioux, E. Caumes, Y. Yazdanapanah

**Affiliations:** 1 APHP, Hôpital Bichat Claude Bernard, Service des Maladies Infectieuses et Tropicales, Paris Diderot University, Paris, France; 2 APHP, Hôpitaux Universitaires Pitié Salpêtrière-Charles Foix, Service des Maladies Infectieuses et Tropicales, Paris, France; 3 Virology Department, APHP-Bichat-Claude Bernard Hospital, Paris, France; 4 AP-HP, Hôpitaux Universitaires Pitié Salpêtrière-Charles Foix, Service de Virologie, et Sorbonne Universités, UPMC Univ Paris 06, CR7, CIMI, INSERM U1135, Paris, France; 5 APHP, Infection control unit, Bichat Claude Bernard hospital, Paris Diderot University, Paris, France; 6 INSERM, IAME, UMR 1137, Paris, France; 7 Univ Paris Diderot, IAME, UMR 1137, Sorbonne Paris Cité, Paris, France; 8 AP-HP, Hôpitaux Universitaires Pitié Salpêtrière-Charles Foix, Bactériologie-Hygiène Hospitalière, Paris, France; 9 Faculté de Médecine P. & M. Curie Paris-6 - Site Pitié, Centre d’Immunologie et des Maladies Infectieuses (CIMI) - E13, Paris, France; 10 APHP- Hôpital Bichat Claude Bernard, Service de Réanimation médicale et Infectieuse, Paris, France; 11 Service de pneumologie et réanimation Département R3S, AP-HP, Hôpitaux Universitaires Pitié Salpêtrière-Charles Foix, unité de Soin de Réadaptation Post Réanimation (SRPR), Paris, France

**Keywords:** Middle East respiratory syndrome coronavirus (MERS-CoV), Pilgrims, Saudi Arabia, Isolation ward, Respiratory tract infection, Legionella

## Abstract

**Background:**

Patients with suspected Middle East respiratory syndrome coronavirus (MERS-CoV) infection should be hospitalized in isolation wards to avoid transmission. This suspicion can also lead to medical confusion and inappropriate management of acute respiratory syndrome due to causes other than MERS-CoV.

**Methods:**

We studied the characteristics and outcome of patients hospitalized for suspected MERS-CoV infection in the isolation wards of two referral infectious disease departments in the Paris area between January 2013 and December 2016.

**Results:**

Of 93 adult patients (49 male (52.6%), median age 63.4 years) hospitalized, 82 out of 93 adult patients had returned from Saudi Arabia, and 74 of them were pilgrims (Hajj). Chest X-ray findings were abnormal in 72 (77%) patients. The 93 patients were negative for MERS-CoV RT-PCR, and 70 (75.2%) patients had documented infection, 47 (50.5%) viral, 22 (23.6%) bacterial and one *Plasmodium falciparum* malaria. Microbiological analysis identified *Rhinovirus* (27.9%), *Influenza* virus (26.8%), *Legionella pneumophila* (7.5%), *Streptococcus pneumoniae* (7.5%), and non-MERS-coronavirus (6.4%). Antibiotics were initiated in 81 (87%) cases, with two antibiotics in 63 patients (67.7%). The median duration of hospitalization and isolation was 3 days (1–33) and 24 h (8–92), respectively. Time of isolation decreased over time (*P* < 0.01). Two patients (2%) died.

**Conclusion:**

The management of patients with possible MERS-CoV infection requires medical facilities with trained personnel, and rapid access to virological results. Empirical treatment with neuraminidase inhibitors and an association of antibiotics effective against *S. pneumoniae* and *L. pneumophila* are the cornerstones of the management of patients hospitalized for suspected MERS-CoV infection.

**Electronic supplementary material:**

The online version of this article (10.1186/s12879-018-3223-5) contains supplementary material, which is available to authorized users.

## Summary

During the 2013–2016 period, 93 patients were managed in two Parisian referral centers for possible MERS-CoV infection. None of them were confirmed as MERS-CoV positive. Seasonal and influenza viruses were the most common pathogens but bacterial pneumonia was also diagnosed.

## Background

Middle East respiratory syndrome coronavirus (MERS-CoV) is a single-stranded positive-sense RNA virus firstly isolated in 2012 in the Kingdom of Saudi Arabia (KSA) [1]. In December 2016, the World Health Organization (WHO) reported 1917 laboratory-confirmed cases of MERS-CoV, and 684 deaths in 27 different countries [2]. MERS-CoV is a zoonotic virus of incompletely elucidated origin. Dromedary camels are suspected of being the reservoir, with bats as a possible origin because they harbor related viral sequences [3–8]. Human-to-human transmission requires close contact with infected people [9]. To date, all reported MERS-CoV cases have occurred in individuals from Arabian Peninsula countries, in travelers returning from this area or traced to an ill traveler.

Each year, during the Muslim Hajj and Umrah pilgrimage, millions of people travel to the Middle East. During this period there is a high risk among pilgrims of acquisition of respiratory tract infections, including MERS-CoV [10]. Although the risk of MERS-CoV acquisition is extremely low in pilgrims and travelers, the consequences of such acquisitions can be dreadful, as illustrated by a South Korean outbreak in which one index case led to 186 secondary and tertiary cases, including 36 deaths [11–13]. Nosocomial epidemic events have also been described in the KSA, where new cases in residents are still reported monthly [13–15]. These reports show that MERS-CoV is mostly characterized by nosocomial transmission and family clusters [13]. Interestingly, it has been shown that cross-transmission can be contained at home as well as in hospital settings when suspected cases are put under contact restriction and enhanced hygiene procedures are applied in combination with rapid testing for MERS-CoV [16].

In France, a national plan was set up in 2012/13 in which reference wards were identified in each region for rapid isolation of suspected cases, with prompt case ascertainment based on epidemiological and clinical characteristics with the help of the national institute of health [17]. Despite this framework, one case of MERS-CoV infection identified in 2013 in a traveler from the Middle East region led to a secondary case after nosocomial acquisition [18]. The clinical symptoms of MERS-CoV infection have low specificity [19]. Therefore the “true” etiology of an acute respiratory syndrome can be overlooked when focusing only on MERS-CoV infection in a suspected case and this may result in a lost opportunity [20]. This is particularly true during the period when people return from the Hajj, where the number of suspected cases increases greatly during a short time.

Here we report our four-year experience in the Paris area on the management and outcome of patients hospitalized in two isolation wards for suspected MERS-CoV infection. We performed a descriptive analysis to better define viruses or bacteria causing infections in this MERS-CoV negative population.

## Methods

This retrospective analysis was carried out in the infectious diseases departments of two university hospitals (Bichat Claude Bernard and Pitié-Salpêtrière) in Paris, France. Both departments are part of the Paris/Ile de France regional plan for the management of contagious emerging infectious diseases in adults. They serve as referral centers for emerging infectious diseases in the Paris area and have isolation wards and dedicated rooms with anterooms and negative pressure. We enrolled patients who had been admitted to these two wards after being classified as possible cases of MERS-CoV infection, as defined by the WHO epidemiological bulletins [21].

In hospitalized patients, epidemiological data were collected: demographic characteristics, travel history, purpose of the travel, contact with animals or sick people and inpatient or outpatient visits during the travel. Also recorded were the nature of the initial symptoms, and the lag time between symptom onset and both the date of departure from the at-risk region, and hospitalization in an isolation ward.

Upon admission to the isolation wards, clinical symptoms and comorbidities were studied, the initial laboratory findings were assessed, and chest X-ray was defined as normal or showing alveolar and interstitial infiltrates.

Clinical management was evaluated in terms of the place of hospitalization, antibiotic treatment, antiviral use and oxygen administration. Microbiological parameters recorded included all bacteriological and virological tests performed during the hospital stay of the patient. Nasopharyngeal specimens were collected for real-time RT-PCR analysis. Respiratory specimens were obtained as soon as possible during the course of the illness (within 21 days after symptom onset). Laboratory confirmation of MERS-CoV infection has been performed on site since 2013, during the opening hours of the local laboratories and in the Pasteur Institute reference laboratory at night and weekends. Confirmation was performed by specific real-time RT-PCR assay, using two different MERS-CoV genomic target sites, the region upstream of the envelope gene and the site of *ORF1* [22]. Film Array Rapid multiplex PCR was performed for simultaneous qualitative detection and identification of multiple respiratory viral and bacterial nucleic acids in nasopharyngeal swabs (FilmArray® Respiratory Panel Biomérieux Lyon France): adenovirus, coronaviruses, human metapneumovirus, influenza A and B viruses, parainfluenza viruses, respiratory A and B viruses*, Bordetella pertussis*, *Chlamydophila pneumoniae*, and *Mycoplasma pneumoniae***.** Bacteria were documented using diverse methods including blood cultures, serology, urinary antigens, sputum and pulmonary samples, respectively. Blood smear for malaria was performed in at-risk travelers. Isolation was maintained until a negative result was obtained for MERS-CoV if the symptoms were more than 4 days old (otherwise a second sample was needed). Duration of isolation and total duration of hospitalization were recorded.

Comparisons were performed using Kruskal-Wallis tests for continuous variables and Pearson’s *χ*^2^ or Fisher’s exact test for categorical variables. Statistical analysis was performed using R (v3.2.0, Vienna, Austria); significance was set at a *p*-value < 0.05.

According to the French Health Public Law (CSP Art L1121–1.1), such an investigation does not require specific informed consent or ethics committee approval because it is a retrospective study without medical intervention.

## Results

### Clinical characteristics

**Fig. 1:**
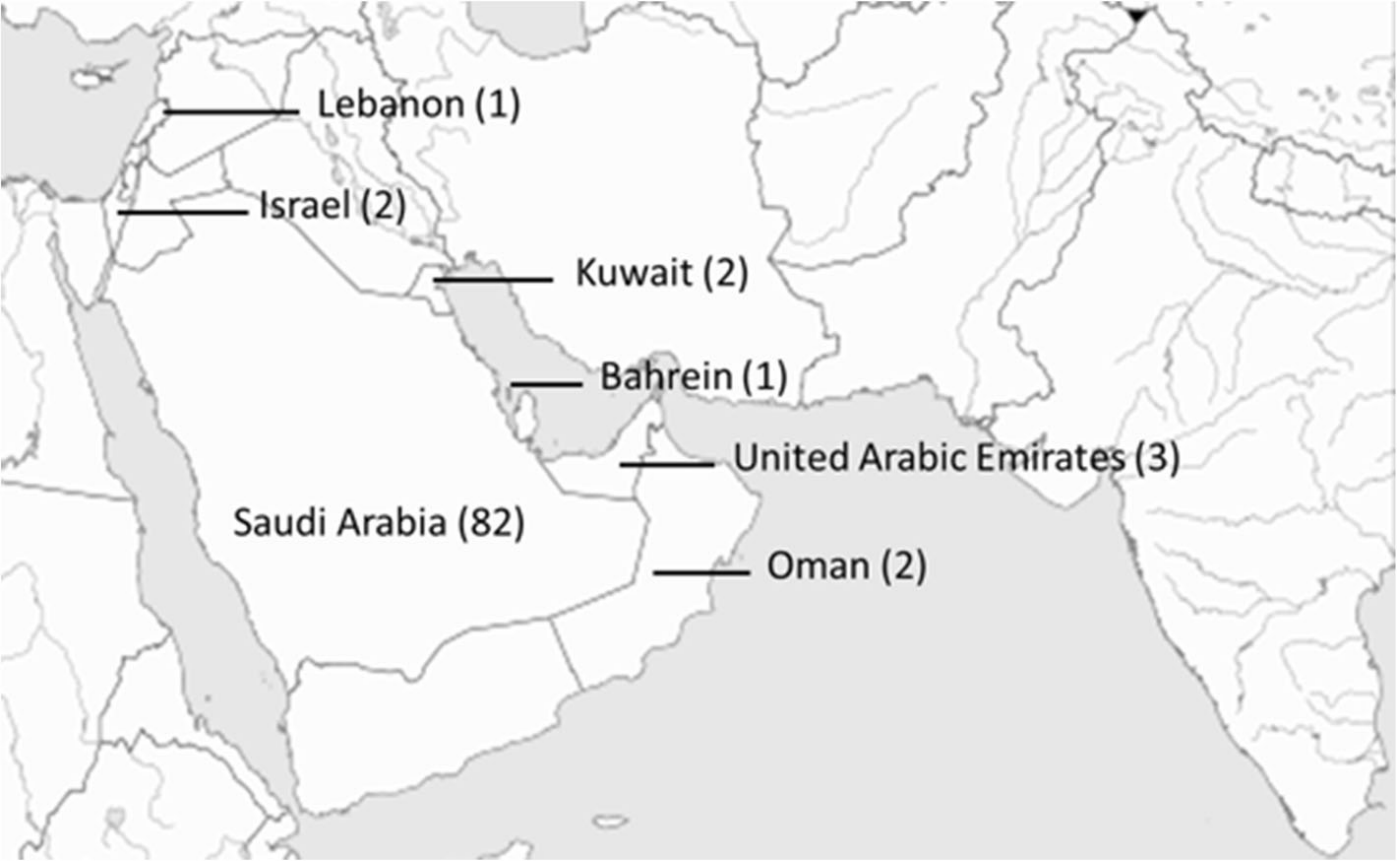
Geographic distribution of the 93 patients prior to their hospitalization in an isolation ward for suspected MERS-COV infection. Adapted from Виктор В https://fr.wikipedia.org/wiki/Fichier:Outline_map_of_Middle_East.svg

**Fig. 2:**
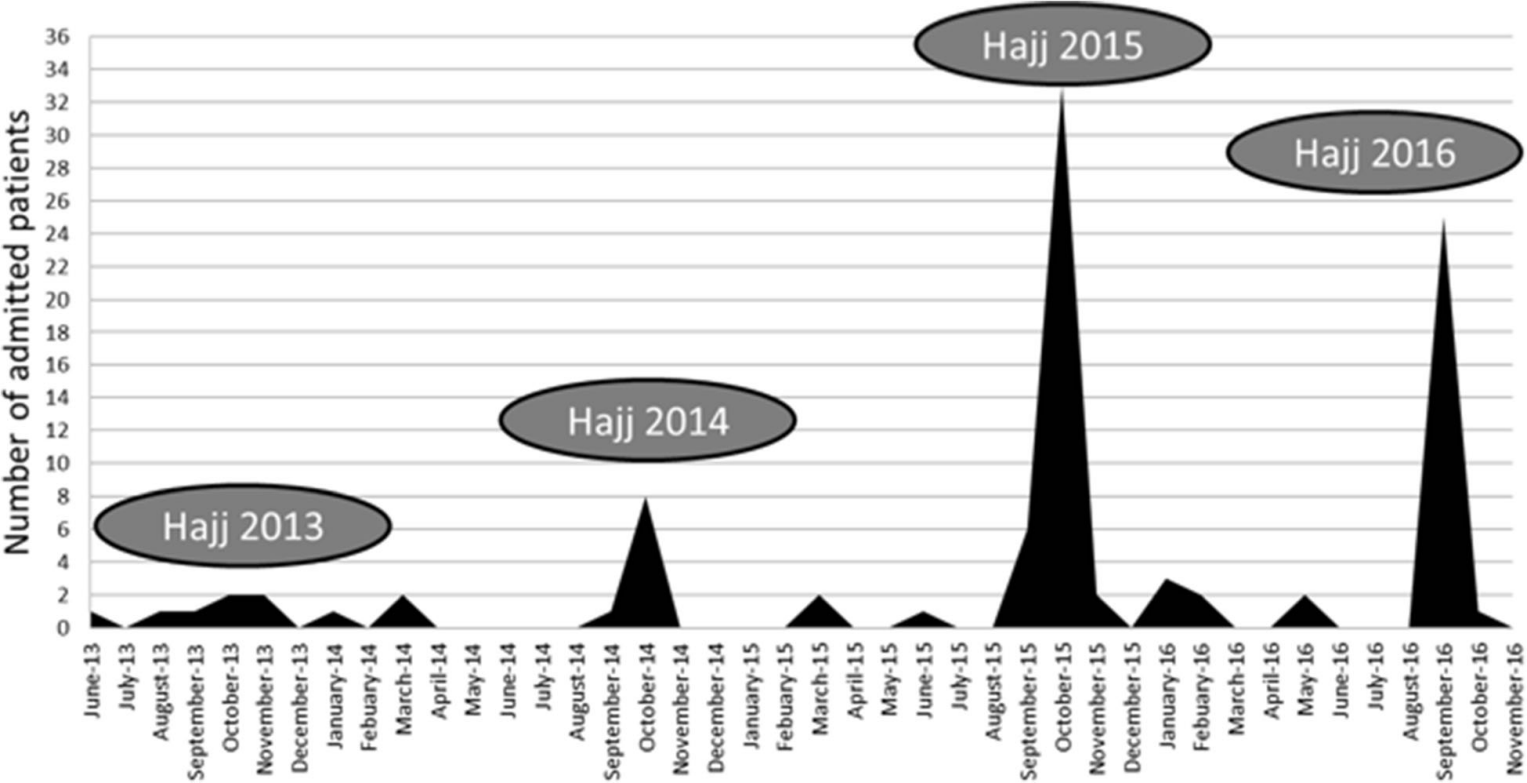
Total number of admissions for suspected MERS-CoV infection according to the time of year, with special reference to the Hajj pilgrimage. The vertical axis represents the number of patients admitted per month. The horizontal axis represents dates. Boxes inside the diagram indicate the annual Hajj pilgrimage

From January 2013 to December 2016, 93 adult patients, classified as possible MERS-CoV cases, were hospitalized in the two participating isolation wards. The male: female ratio was 1.1 and the median age was 63.4 years (interquartile range; IQR, 56–71.5). Of 82 (88.2%) patients who were returning from the KSA, 74 (90.2%) had travelled for the pilgrimage, two (2.1%) for professional reasons, and four (4.9%) for tourism; two (2.4%) were KSA residents (Fig. 1). There was an obvious seasonal trend with a major annual increase in the number of admitted patients during the annual Hajj period (Fig. 2). The median travel duration was 23 days (IQR = 17–27). The median lag time between the first symptoms and admission to the isolation ward was 8.2 days (IQR, 0–28). The median lag time between arrival in France and admission to the isolation ward was 2 days (IQR, 1–5 days), and only 19 (20.4%) patients had the first symptoms after their arrival in France.

The first symptoms described by the patients were cough (*n* = 62, 67%), influenza-like symptoms (*n* = 18, 19%), and dyspnea (*n* = 6, 6%). Other symptoms were diarrhea (*n* = 2, 2%), fever (*n* = 2, 2%), vomiting (*n* = 1, 1%), general illness (*n* = 1, 1%) and headache 1(1%). Thirty-four (36.5%) patients had consulted a physician in the at-risk countries and nine (9.7%) had prior hospitalization in these countries.

Close contact with ill travelers with respiratory symptoms during travel was found in 43 (46.7%) patients. Exposure to dromedary camels or contaminated camel milk or meat was found in five (5.4%) patients.

Initial clinical findings of patients admitted to isolation wards are reported in Tables 1 and 2. The most common signs were cough (95.7%), with 63 (70.7%) patients having sputum production, and 26 (29.3%) dry cough. Pulmonary auscultation revealed crackles in 61 (65.5%) patients, bilateral in 25 (26.9%). Ten (11%) patients required oxygen therapy at initial evaluation. Other clinical symptoms are listed in Table 2. Ten patients (11%) were admitted to intensive care directly (*n* = 6) or after evaluation in isolation wards (*n* = 5).

**Table 1:** Clinical and travel characteristics of 93 patients with possible MERS-CoV infection hospitalized during 2013–2016

Characteristics		
Sex ratio M/F		49/44
		Median (IQR)
Age (year)		63.4 (56–71.6)
Temperature (°C)		38.4 (37.2–39)
Systolic blood pressure (mm Hg)		133 (120–148.5)
Diastolic blood pressure (mm Hg)		77 (66.5–83.5)
Heart rate (beats/min)		91 (81.5–108-5)
Respiratory frequency/min		20 (18–24)
Oxygen saturation		96 (93–98)
NYHA score		2 (1–3)
Duration of travel (days)		6.5 (4–12)
Time of illness (days) median (IQR) 6.5 (4–12)		
Reason for travel	N	%
Pilgrimage	74	79.6
Business	4	4.3
Tourism	11	11.8
Resident	4	4.3
Duration of travel (days) 23 (17–27)		

*New York Heart Association* (*NYHA*) Functional *Classification*, *KSA* Kingdom of Saudi Arabia, *UAE* United Arab Emirates

**Table 2:** Presenting symptoms and laboratory findings on admission in 93 patients with possible MERS-CoV infection hospitalized during 2013–2016

Symptoms	N	%
Cough	89	95.7
Fever (> 38 °C)	61	65.6
Lung crackles	61	65.6
Rhinorrhea	42	45.2
Myalgia	30	32.3
Headhache	26	28.0
Thoracic pain	22	23.7
Diarrhea	20	21.5
Abdominal pain	13	14.0
Vomiting	12	12.9
Nausea	11	11.8
Hemoptysis	9	9.7
Laboratory tests (n)	Median	IQR
CRP mg/dL (81)	122	41–247
WBC G/L (90)	9.295	6.45–12.325
Neutrophils G/L (75)	8.285	4.61–10.26
Lymphocytes G/L (63)	1.300	0.93–2.02
Platelets G/L (89)	268.	179–320
Serum creatinine μmol/L (89)	78.3	57–87

*CRP* C-reactive protein, *WBC* white blood cell count

Seventy-five (80.6%) patients had underlying medical conditions with a median of 2 (1–3) different comorbidities such as hypertension (*n* = 57, 61.3%), chronic respiratory diseases (*n* = 22, 23.6%), chronic cardiac disease (*n* = 21, 22.6%), or obesity (*n* = 19, 20.4%). Nine patients (9.7%) had a history of neoplastic or hematological disease, six (6.4%) were receiving corticosteroids, and six (6.4%) immunosuppressive drugs.

### Chest X-ray evaluation and laboratory results

Initial chest X-ray was available for 90 patients (96.7%), and findings were abnormal in 72 (80%). Thirty-nine of these 72 (54.1%) exhibited alveolar opacities with 4 (10.4%) bilateral localization, 20 (27.7%) interstitial syndrome, including 12 (60%) with bilateral localization, and 13 (18%) alveolar and interstitial syndrome, with bilateral localization in 9 (69.2%) (Additional file 1: Figure S1).

Laboratory findings are reported in Table 2. On admission, the median C-reactive protein was 122 mg/dL (21–247) and above 50 mg/dL in 57 (70.3%) patients. Neutrophil count was increased in 15 patients. Median neutrophil count was 8.285 G/L (4.61–10.26).

### Therapeutic management

Among the 93 patients, 81 (87.1%) initially received antibiotic therapy: 63 (69.3%) antibiotic combinations (AC), and 18 (19.4%) a single antibiotic. AC were administered for a median duration of 48 h (IQR, 24–116), and were secondarily switched in 48 (76.2%) cases and discontinued in 15 (23.8%). AC were third-generation cephalosporin and macrolides in 55 patients (87.3%), aminopenicillin and macrolides in three patients and third-generation cephalosporin and fluoroquinolone in four patients. An AC was maintained in 10 (19.2%) cases, with aminopenicillin–spiramycin in six and quinolones plus spyramicin or rifampicin in four. Thirty-eight (60.3%) patients were switched to a single antibiotic as amoxicillin-clavulanate in 12 (31.5%), third-generation cephalosporin in eight, amoxicillin in seven, spiramycin in six, levofloxacin in three, and piperacillin-tazobactam and doxycycline each in one.

Single antibiotics initially prescribed were amoxicillin-clavulanate in nine patients (50%), amoxicillin in five, or third-generation cephalosporin in four. This antibiotic was maintained throughout treatment in 14 cases and switched to oral amoxicillin-clavulanate in four cases, with no discontinuation in the 48 first hours of treatment.

### Empirical antiviral treatment with neuraminidase inhibitors

Oseltamivir was given to 35 patients (37.6%) for a median duration of 120 h (24–120). It was discontinued after 48 h in 12/35 (34.3%) patients, between 48 h and 5 days in 4 (11%) patients and kept for the entire treatment duration in 19 (54.3%) patients.

Oxygen therapy was required for 42 patients (45.6%), with a median maximal flow of 3 L/min (IQR = 2–4). Five (5.4%) patients had an oxygen flow of more than 6 L/min and were transferred to intensive care.

### Microbiological documentation

None of the patients hospitalized for suspected MERS-CoV inhibition was found to be positive on MERS-CoV PCR. A microbiological etiology was identified in 70 (75.4%) patients (Table 3).

**Table 3:** Pathogens in the 70 patients with microbiological data

	Monomicrobial infection	Mixed Infection							
	No other microorganism	Human Coronarovirus	*Influenza* A	*Influenza* B	*K. pneumoniae*	*S. pneumoniae*	*S. mitis*	*S. aureus*	Total
Non MERS Coronavirus_	3		1						4
*Influenza* A	13			1		2	1		17
*H. influenzae*	1		1						2
*L. pneumophilia*	6		1						7
Rhinovirus	16	2	3		1	2		1	25
*S. paratyphi*	1								1
*Streptococcus A*	1								1
*Coxiella burnetti*	1								1
HSV-1	1								1
Metapneumovirus	2								2
*Infleunza* B	2								2
*P. falciparum*	1								1
Parainfluenza	2								2
*S. pneumoniae*	3								3
Negative	23								23
Total	76	2	6	1	1	4	1	1	92

A viral infection was documented in 47 patients (50.3%), including 26 (37%) with human rhinovirus (HRV) and 25 (35.6%) influenza virus. For the latter, each type of virus was identified in approximately half of the cases.

A bacterial infection was documented in 22 patients (23.6%), the most common etiologies being *Legionella pneumophila* and *Streptococcus pneumoniae* in 10 patients (31.8%) each. Patients with Legionnaires’ disease (LD) had more chronic cardiac disease and were more immunosuppressed than other patients (data not shown). Finally, those with LD had a longer duration of hospital management (11 days vs. 4 days, *p* = 0.001). *Plasmodium falciparum* malaria was diagnosed once.

A mixed infection was documented in 16 (17.4%) patients, 43% being mixed viral infections and 56% mixed virus-bacterial infections.

Empirical antibiotic therapy was prescribed to 21/22 patients with a documented bacterial infection, and was effective in 21 against the bacterial strains secondarily documented during the course of the disease. Among the 47 patients with a documented viral infection, 39 had received empirical antibiotic therapy. Lastly, of 23 patients without any documentation 21 had received empirical antibiotic therapy. Of the 25 patients with documented influenza virus, 13 (52%) had received neuraminidase inhibitors (oseltamivir).

### Isolation precautions

**Fig. 3:**
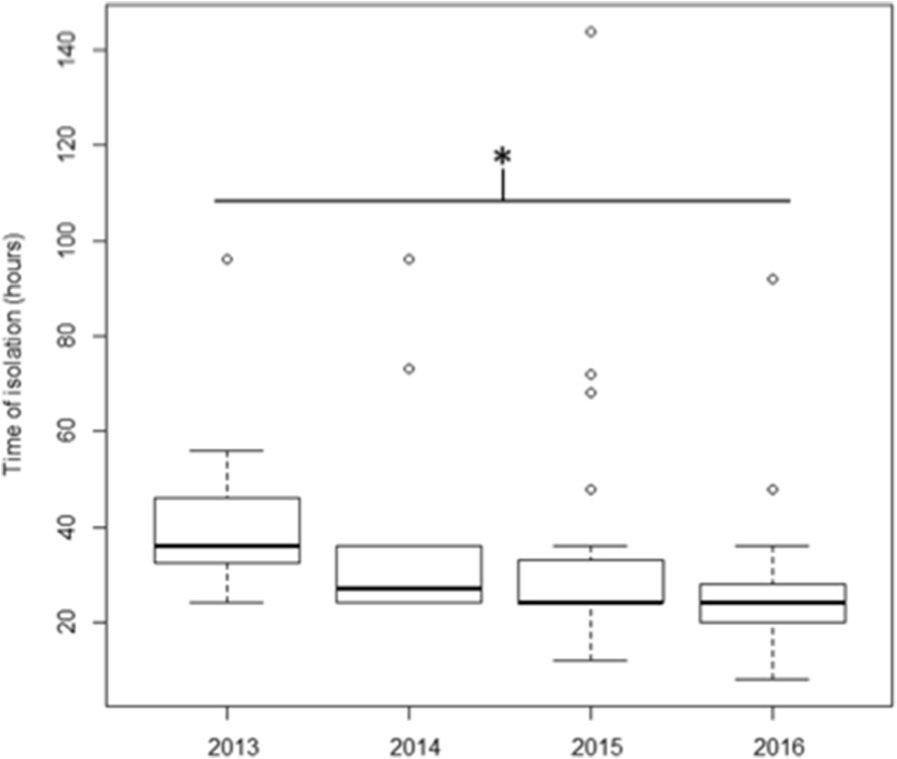
Duration of isolation precautions in the isolation ward from 2013 to 2016. Each boxplot represents the duration of isolation for patients hospitalized per year, horizontal bars in the boxes represent the median, horizontal bars outside the boxes represent interquartile range (IQR), and circles represent outliers. The asterisk and the black bar represent *p*-valued under 0.05 using the Kruskal-Wallis test

The duration of isolation was calculated from the initial confinement triggered by the suspicion of MERS-CoV infection, upon the removal order by the physician following receipt of the negative result of the MERS-CoV analysis. The median duration of isolation precautions was 24 h (IQR, 24–32.5) and ranged from 8 to 144 h. The duration of isolation decreased significantly over the years from a median of 36 h in 2013 to 24 h in 2016 (*p* < 0.01) (Fig. 3). Patients were hospitalized in the isolation ward for a median of three days (IQR 2–5.22), and the total management duration from alert to hospital discharge was four days (IQR 3–8).

### Mortality

Two of the 93 (2.1%) patients died of malignant flu and with a *Staphylococcus aureus* related prosthetic heart valve infective endocarditis, respectively.

## Discussion

During a four-year period including four Hajj and Umrah pilgrimages, our two centers managed 93 patients for possible MERS-CoV infection. None of the patients returning from endemic countries and classified as possible cases were confirmed as MERS-CoV positive. Seasonal viruses and influenza viruses were the most common pathogens identified, but life-threatening bacterial pneumonia was also diagnosed.

Influenza viruses were found in 35% of the microbiologically documented patients, which is consistent with other results showing prevalence ranging from 13 to 64% for patients with suspected MERS-CoV infection [23–25]. In an Iranian study, influenza prevalence was around 10% in pilgrims with upper respiratory tract infections [26]. These results suggest that empirical oseltamivir therapy should be initiated in patients admitted to the isolation ward for suspected MERS-CoV infection. This antiviral treatment should be discontinued when PCR results prove negative for influenza. Moreover, these results illustrate the importance of preventive flu vaccine prior to pilgrimage and in all travelers to areas such as those where MERS-CoV is endemic [27].

In our study, 31.4% of the patients were positive for HRV, compared with 10 and 23% in two other studies [10, 28]. This is also in agreement with a study in French pilgrims that demonstrated acquisition of HRV during the trip using a pre- and post-travel routine screening [10]. HRV involvement in acute respiratory infection remains controversial, and further studies on phylogenetic and epidemiological characteristics are necessary to clarify the clinical impact of this virus [29].

Altogether, about one-quarter of our patients had species infections associated or not with viral infection. The two most frequently documented bacteria were *S. pneumoniae* and *L. pneumophila*. Pneumococcal disease was diagnosed in seven patients; all had received empirical antibiotics effective against *S. pneumoniae*. It has been showed that *S. pneumoniae* carriage increases in French pilgrims during the Hajj [10], whereas vaccine coverage was low (7%) in 300 French Hajj pilgrims [30]. Therefore, pneumococcal vaccination should be proposed to at-risk persons before the Hajj pilgrimage.

Seven patients presented *L. pneumophila* infection. They all had previous underlying conditions with greater chronic cardiac disease and more immunosuppressed status. At initial evaluation, they presented with more headache, increased respiratory frequency, more intense dyspnea and a more prolonged duration of hospitalization. None of them died, but two patients required admission to intensive care units. LD has already been reported as an alternate diagnosis of MERS-CoV infection in a series of 77 suspected patients where 22 had positive results for alternative respiratory pathogens including two LD [23].

Pilgrims during the Hajj frequently stayed in accommodation opened only during the pilgrimage. *Legionella spp.*, the causative bacterium, is found naturally in fresh water and can contaminate hot tubs and cooling towers of air conditioners. The conditions of accommodation could partly explain LD acquisition during travel, as recently reported by the European Centre for Disease Prevention and Control, with more than 30 laboratory-confirmed cases of LD diagnosed in travelers from the European Union to Dubai since October 2016 [31], representing a significant increase over the average incidence. The probability of LD in patients with possible MERS-CoV infection highlights the need for an initial antibiotic combination with anti-Legionella efficacy, and local surveillance in pilgrim facilities.

One patient had a *P. falciparum* infection. This patient had made a stopover in Mali without antimalarial chemoprophylaxis during his return trip to France. Antimalarial treatment should be proposed to every patient with suspected MERS-CoV infection when they come back from a malarial endemic region.

A few sporadic travel-associated MERS-CoV cases have been reported outside the Arabian Peninsula, with 26 different countries involved, including Republic of South Korea (RSK). Regarding the very high number of travelers who stay in at-risk countries, especially during the Hajj, MERS-CoV transmission remains rare. In a recent MERS-CoV surveillance study conducted in the KSA during the annual Hajj pilgrimage, of 888 people screened during September 2015, none tested positive for MERS-CoV [32]. This is also supported by the absence of person-to-person MERS-CoV transmission during the mass gatherings at the Hajj in 2013–15 [33].

Nosocomial transmission of MERS-CoV, as in the KSA and RSK could be contained if every suspected case is managed with strict isolation precautions. This organization is implemented in France, with an alert system and referral departments trained and organized to receive possibly infected patients. This organization requires transfer of patients from the first care facility, where the suspicion arises, to the referral isolation ward. This might delay appropriate management of patients with acute respiratory infection and result in a lost opportunity as already detailed in one of our two case [20]. During our study period, patients with possible MERS-CoV infection were mostly (78.5%) admitted during short time periods linked to pilgrimage periods (Fig. 2). Moreover, the low specificity of the clinical symptoms [19] could lead to medical mistakes, and life-threatening diseases not fully covered by empirical antimicrobial treatment. Laboratory tests are limited in this setting excluding blood cultures. In our study, four diagnoses were likely delayed and not covered by the empirical treatment prescribed: Q fever, malaria, *S. aureus* endocarditis and HSV-1 stomatitis.

Our study had some limitations: it was retrospective and some data could not be obtained from all patients. There were only two MERS-CoV documented cases in the North of France [18]. Since these two cases, no new cases were described, but every year thousands of pilgrims and travelers return from endemic countries with respiratory syndrome, which engages the MERS-CoV procedure and leads to the transfer of these patients to isolation wards for diagnosis exclusion. Taking a control group with French non-traveler patients hospitalized for Respiratory infection seemed inappropriate. In a similar way, using travelers hospitalized for Respiratory tract infection did not seemed relevant because the only difference would be the travel destination as shown in travelers during the H1N1 pandemic [34]. Our Knowledge of MERS-CoV has grown over the years, and during this time management of possible cases has improved in terms of empirical treatment, which could have influenced our results. MERS-CoV RT-PCR was made available locally in 2013, thus reducing the time of diagnosis, which was also influenced by the time and day of the patient’s arrival linked to the local laboratories opening hours.

## Conclusions

Empirical antiviral treatment with neuraminidase inhibitors as well as antibiotic treatment effective against infection due to *L. pneumophila* and *S. pneumoniae* should be considered in any patients with suspected MERS-CoV infection. Altogether, our results argue for specific infectious disease units dedicated to emerging infectious disease management, but also for the development of new tools to simplify and expedite the diagnostic process in patients with suspected MERS-CoV infection in order to interrupt isolation procedures earlier.

## Additional file

Additional file1: **Figure S1.** Initial Chest X-Ray results of the 93 patients. (TIF 101 kb)

## Abbreviations

HSV-1: Herpes simplex virus1
IQR: Interquartile range
KSA: Kingdom of Saudi Arabia
LD: Legionnaires’ disease
MERS-CoV: Middle East respiratory syndrome coronavirus
RSK: Republic of South Korea
RSV: Rhinovirus
RT-PCR: Real Time Polymerase Chain Reaction
UAE: United Arab Emirates
WHO: World Health Organization
