# Supplementary figures and images for: Clinical management of respiratory syndrome in patients hospitalized for suspected Middle East respiratory syndrome coronavirus infection in the Paris area from 2013 to 2016
Source: BMC Infect Dis. 2018 Jul 16;18:331. doi: 10.1186/s12879-018-3223-5 (PMC6048819; doi:10.1186/s12879-018-3223-5)

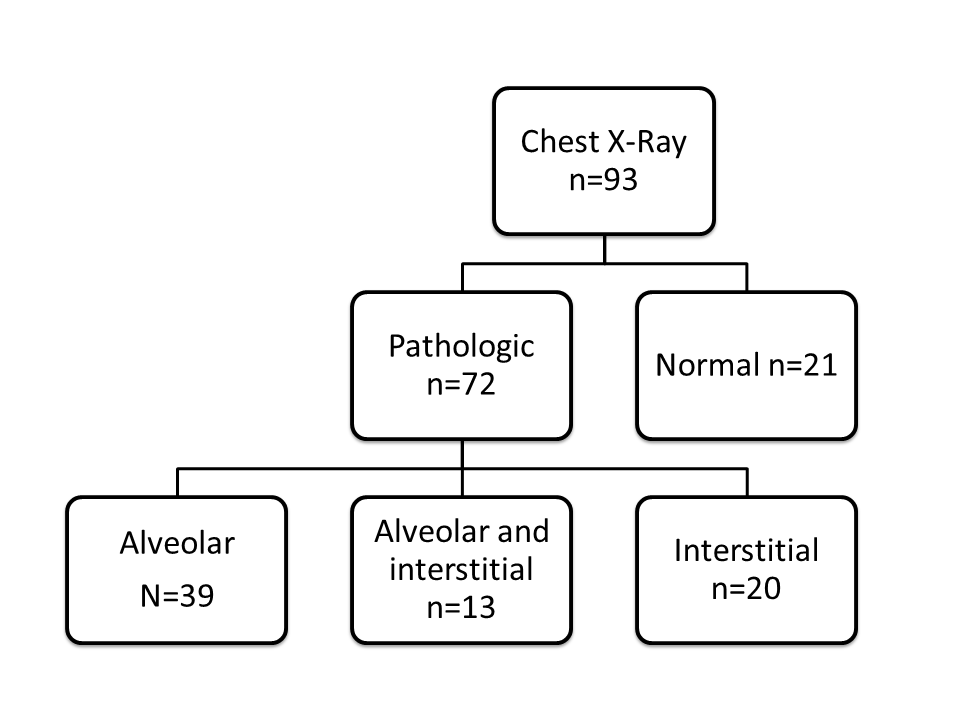

Supplement: Supplementary file 1 — Figure S1. Initial Chest X-Ray results of the 93 patients. (TIF 101 kb) [file 12879_2018_3223_MOESM1_ESM.tif]
